# Bionic Vision-Based Intelligent Power Line Inspection System

**DOI:** 10.1155/2017/4964287

**Published:** 2017-01-19

**Authors:** Qingwu Li, Yunpeng Ma, Feijia He, Shuya Xi, Jinxin Xu

**Affiliations:** Key Laboratory of Sensor Networks and Environmental Sensing, Hohai University, Changzhou 213022, China

## Abstract

Detecting the threats of the external obstacles to the power lines can ensure the stability of the power system. Inspired by the attention mechanism and binocular vision of human visual system, an intelligent power line inspection system is presented in this paper. Human visual attention mechanism in this intelligent inspection system is used to detect and track power lines in image sequences according to the shape information of power lines, and the binocular visual model is used to calculate the 3D coordinate information of obstacles and power lines. In order to improve the real time and accuracy of the system, we propose a new matching strategy based on the traditional SURF algorithm. The experimental results show that the system is able to accurately locate the position of the obstacles around power lines automatically, and the designed power line inspection system is effective in complex backgrounds, and there are no missing detection instances under different conditions.

## 1. Introduction 

The reliability and stability of the power system are essential for the social development. And the distance between the high voltage power lines and the external obstacles must be farther away compared to the safety distance. The most common external obstacles of the power lines are buildings and plants. Growth of particularly the growing plants, such as trees, is irregular and not controlled by the time and environment. It will lead to the abnormal discharge when the plants are close to the power lines, and then the power lines will show the phenomenon of fever and fracture. So the growth of plants around the power lines is a hidden threat to the power lines. Therefore, complex terrain and bad environment of the growing plants make the power line inspection tedious, expensive, time-consuming, and dangerous.

At present, there have been some power line inspection methods put into practical work, which involves four main directions: foot patrol [1], manned aerial vehicles [2], climbing robots [3, 4], and unmanned aerial vehicles (UAVs) [5].

*(A) Foot Patrol Inspection*. The components of a foot patrol inspection team were introduced in [1]. Usually, a team of two people walk from pylon to pylon to inspect the power lines in the foot patrol inspection. Visual inspection is carried out with the help of binoculars and sometimes with infrared radiation and corona detection cameras. Such an inspection can be highly accurate only when the surfaces of power line equipment can be well seen from the ground. Recently, foot patrol inspection requiring the experience of staff has taken the main responsibility for the detection of fault in the power lines, which will take lots of time and labor. However, it is not easy to find hidden faults promptly by this means. Many dangerous areas are blind through this kind of inspection method.

*(B) Manned Aerial Vehicles*. Helicopter-assisted inspection is also becoming common for power line inspection. The team is usually made up of three people, respectively: the pilot, the inspector, and the recorder. In the helicopter over the power lines, the staff will have a wider field of vision and faster inspection speed, and they can get more accurate data with the help of color, infrared radiation, and corona detection camera. There have been several works using this method, focusing on the sensor fusion, as in [6–8]. However, given the high costs involved in such inspections, their use should only be rented in quick inspection of large networks or in places where it is difficult to access land. In this way, the staff is not necessary if the aircraft can automatically collect power line data; therefore other alternatives have begun to be explored such as climbing robots or UAVs.

*(C) Climbing Robots Inspection.* Compared with the foot patrol inspection and the manned aerial vehicles, inspection conducted by climbing robots would be safer, less tedious, objective, and much faster. But this kind of inspection method also has many shortcomings, and how to overcome these shortcomings is the direction of the research. Meanwhile, with the electromagnetic field of the power lines, the electronics and sensors on the robot have to be protected, as pointed out in [9, 10]. 

*(D) Unmanned Aerial Vehicles (UAVs)*. Along with the development of the UAV technology, the reconnaissance equipment of UAV has been more and more complex, developing from the early days of the photoelectric camera to optoelectronic platform. The UAV provided a new way in power line inspection, which not only reduces the safety risks and costs but also has a more flexible mode of mobility, enables a wider range of inspections, and gains more accurate data information. Many studies have focused on the integration of multiple images collected by UAVs to determine the power lines status, especially in the fusion of infrared and visible images, as in [10, 11]. According to the information of various types of images, it is easy to find hidden internal faults of power lines promptly, but it is unable to figure out the threats of external obstacles to power lines.

In this paper, we will tackle the mentioned challenges by proposing a new power line inspection system based on the human visual system model for the detection of external obstacles of the power lines. The system can not only inherit many advantages of traditional UAV inspection system but also precisely calculate distance information between the external obstacles and the power lines. The rest of our work is organized as follows: Section 2 briefly describes the characteristics of human vision.  Section 3 gives the description of the UAV inspection system.  Section 4 introduces the processing algorithm in the system based on the human visual system model.  Section 5 presents the overall evaluations and discussions of the system in practice.  Section 6 draws conclusions and proposes future work.

## 2. Human Visual Mechanism and Information Processing Model

After a long period of evolution, biological visual systems develop a strong ability for sensing the world, and they help people's work and life in a variety of forms [12]. In [13], a biological hierarchical model based underwater moving object detection method was presented. In [14], a fly visual perception mechanism based small target detection method was proposed. These two methods make use of the advantages of biological vision in a given environment and have achieved good results in image processing.

Human visual system has some basic properties. Firstly, human vision can always capture the interesting things in a complex environment. In view of this feature, many image segmentation research works have been carried out, as in [15, 16]. Secondly, binocular vision enables human to obtain the depth information of things. Many 3D information research works have been carried out based on binocular vision, as in [17].

### 2.1. Human Visual Attention Mechanism

Human beings can rapidly and accurately identify salient regions in their visual fields. Human vision has such characteristics in terms of color, texture, shape, and brightness. People are always used to paying attention to bright colors, special textures, strange shapes, and high brightness areas immediately in a complex environment. With the improvement of the theory of visual saliency, salience detection is one of the research focuses in computer vision, which simulates human visual attention and vision information processing mechanisms to build saliency computation models. Simulating such ability in machine according to the actual task demand is critical to make machine handle visual content like humans.

### 2.2. Binocular Vision

Human beings perceive and learn the 3D information of the things around them by human visual system. In this process, binocular vision plays an important role in obtaining the depth information of things. Images of the same target are collected in the left and right eyes from different perspectives. And then our brains determine the depth of the target through the parallax in the left and right images. Nowadays, binocular vision technology has been widely used in computer graphics, cognitive psychology, artificial intelligence, and many other fields, and it has successfully provided preferable solutions to many challenging problems in aforesaid fields many times. Binocular vision can be applied to the industrial noncontact detection field for its outstanding advantages such as simple structure, convenient operation, precise measurement, and high speed. It is usually composed of two CCD cameras in structure and can perfectly accomplish the task of distance measurement in some special occasions.

Inspired by the above aspects of visual mechanisms in the eyes of human beings, a power line inspection system based on the human visual system model is proposed in this paper. In the proposed system, the human visual attention mechanism is used to complete the power line segmentation work, and binocular visual model is used to measure the distance between the obstacles and the power lines.

## 3. UAV System

The whole UAV system contains many subsystems [5]:

1. The ground control station (GCS) for accommodating the system operators and the interfaces between the operators and the rest of the aerial system
2. The quadrotor of carrying the payload
3. The system of communication between the quadrotor and the GCS; it transmits controlling inputs to the quadrotor and returns the information from the payload and other data from the aircraft to the GCS

**Figure 1:**
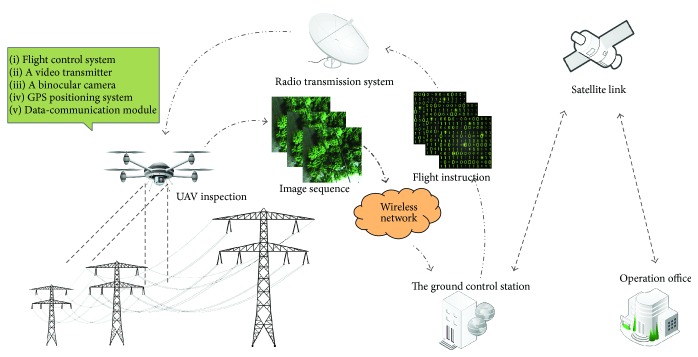
The architecture of the UAV systems for power line inspection.

The hardware system finishes shooting and returns the binocular vision in image sequences under the control of the staff; Figure 1 shows the overall deployment of the UAV systems along with their communication and control solution. In order to collect image information more flexibly, we chose the quadrotor helicopter as the main tool. There are many other modules in the quadrotor helicopter, including flight control system, payload, GPS positioning system, and data-communication module.

### 3.1. Structure of Quadrotor Helicopter

The quadrotor helicopter is a good choice for the power line inspection owing to its good performance in flight. Furthermore, its original small body can be easily adjusted to the practical needs accordingly. When the quadrotor helicopter is used for power line inspection, it will be equipped with a video transmitter, a binocular camera, a GPS, and a data-communication module, and each subsystem has its own function given as follows:

1. The input video is sampled at the video transmitter, and code stream is sent to channel after compression.
2. The binocular camera shoots the power lines through left and right eyes at the same time, and the image sequence will be passed to the video transmitter.
3. When the quadrotor helicopter is flying in manual flight, it attempts to maintain the current altitude and GPS location.
4. Data-communication module contains two information transmission channels, including radio transmission and wireless network. Flight instructions are transmitted by radio transmission and image sequence is transmitted by wireless network.

### 3.2. Ground Control Station

The GCS is generally moving around the power line area and serves as an information hub between the operation office and the quadrotor helicopter. Due to the limited control of the quadrotor helicopter, staffs generally move with the GCS. The GCS includes flight command console, information processing system, and charging system; each subsystem has its own function as follows:

1. Staffs can make flight instructions according to status of the quadrotor helicopter in the command console.
2. The information processing system records the image sequence and location of the quadrotor helicopter.
3. The charging system provides charging service for the quadrotor helicopter.

The staff can get the flight information and status of the quadrotor helicopter from the command console. During the inspection process, the staff controls the quadrotor helicopter flight along the direction of the power lines, and the console shows image processing information and geographic location simultaneously.

## 4. Power Line Inspection Method

**Figure 2:**
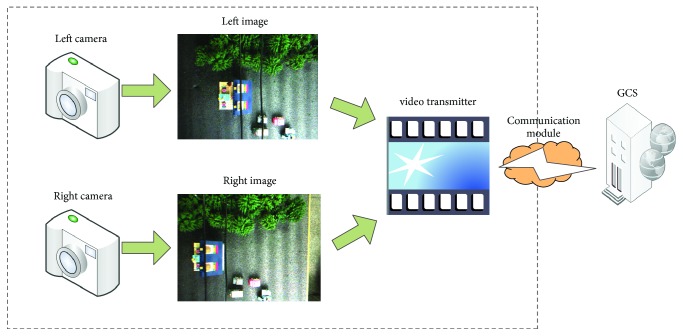
The architecture of visual system scheme.

As given in Figure 2, the visual system includes a binocular camera and it shoots the image sequence over the power line. The video transmitter and the data-communication module together send the image sequence to the GCS, and the visual processing algorithm is implemented in GCS.

**Figure 3:**
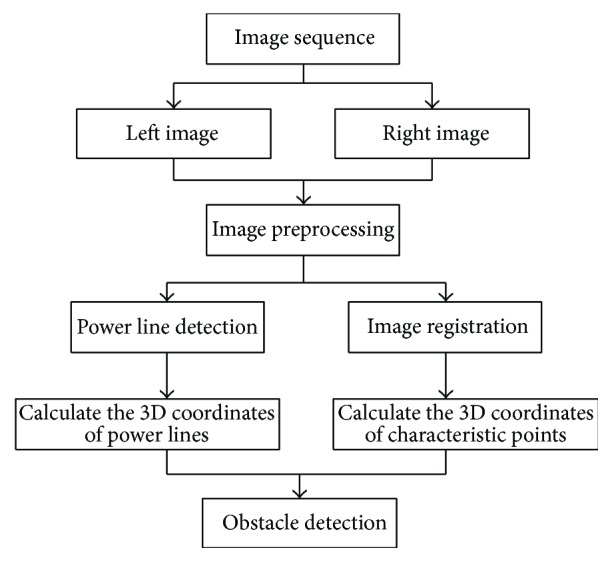
The processing chart of the visual system.

The visual system contains a number of image processing modules: image preprocessing, power line detection, image registration, and obstacle detection, as defined in [18, 19], and the processing of visual system is shown in Figure 3. According to the actual power line distribution, we established the power line model to obtain a more complex background environment so as to check whether our system can sustain better applicability in the real work. We take the single frame images, respectively, selected from the left and right eyes as an example in the system processing. In actual processing, the successive image frames do not contain the same content, and the system processes all the frame images in turn.

### 4.1. Image Preprocessing

**Figure 4:**
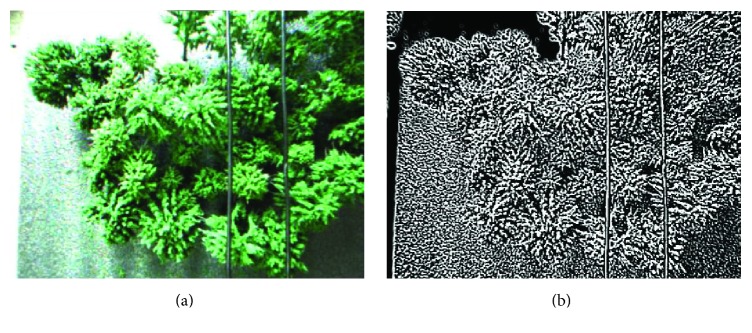
Image preprocessing. (a) Original color image. (b) Preprocessed image.

Assume that the left eye image is *T*_*z*_ and the right eye image is *T*_*y*_. The image preprocessing contains image gray transform and edge detection. Firstly, the system transforms RGB images *T*_*z*_ and *T*_*y*_ into gray images *H*_*z*_ and *H*_*y*_. Secondly, the system detects image points belonging to edge. There are many common methods of edge detection; each method has its own advantages and disadvantages. However, most of them have poor stability and lots of background noises when applied to a complex environment. Therefore, to deal with this situation, the DoG (Difference of Gaussian) edge detection was used in this paper. Lots of experiments in [20, 21] have proven that DoG has good stability and highlights the edge of the power line in the complex background. The edge detection results are shown in Figure 4. DoG is the results obtained by subtracting the Gauss function in different parameters, and the image after being processed by DoG is defined as

(1) DDoGx,y=12π1σ12e−x2+y2/2σ12−1σ22e−x2+y2/2σ22×H=Gx,y,σ1−Gx,y,σ2×H,

where *σ*_1_ = 0.6, *σ*_2_ = 0.9, *H* is a gray scale image, and a sliding filter is defined for the image by the symbol “×.” The preprocessed images are depicted in Figure 4 and are defined as *D*_*z*_ and *D*_*y*_.

### 4.2. Power Line Detection

**Figure 5:**
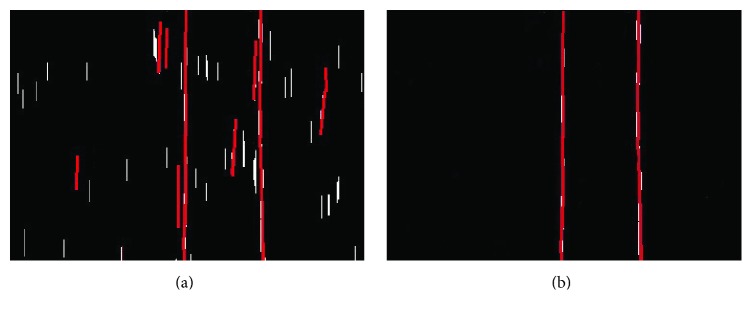
Power line detection. (a) Morphological processing results. (b) Detection results.

The power lines are detected based on the human visual attention mechanism. The human eyes will always notice something special in the image immediately, such as bright colors and unique edges of things [22, 23]. According to the characteristics of the human eyes, a new method is proposed to detect the power lines through liner edges; and the result of the proposed method is shown in Figure 5. The method below is to judge whether there are power lines.

(1) Because the quadrotor helicopter's flight direction is parallel to the direction of the distribution of power lines, power lines are vertically distributed in the image. Firstly, we dilate and erode the image by linear structural factors several times, and the dilation and erosion are defined separately as follows:

(2) dilation:  Y=E⊕B=y:By∩E≠Φ,erosion:  X=E⊗B=x:Bx⊂E,

where *B*(*x*) are structural factors and *E* is the work space. In the choice of the direction of the linear structure, we first use the same length linear structure with a plurality of directions varying from 0° to 180° to process the image and then detect the results and keep the image with the maximum remaining pixels as a target for the next step. When the structure factor is relatively short, the image will only retain power lines after a great deal of dilation and erosion.

(2) As for detecting line segments in the image, line segments in the same slope range are restored to a straight line. Due to the power line throughout the whole image, we can detect power lines through the distribution of linear connected domains.

(3) Regarding recording coordinates of power lines in left and right image, we will get {(*x*_*dz*1_, *y*_*dz*1_),…, (*x*_*dzn*_, *y*_*dzn*_)} and {(*x*_*dz*1_′, *y*_*dz*1_′),…, (*x*_*dzn*_′, *y*_*dzn*_′)} from the left image and {(*x*_*dy*1_, *y*_*dy*1_),…, (*x*_*dyn*_, *y*_*dyn*_)} and {(*x*_*dy*1_′, *y*_*dy*1_′),…, (*x*_*dyn*_′, *y*_*dyn*_′)} from the right image.

### 4.3. Image Registration

**Figure 6:**
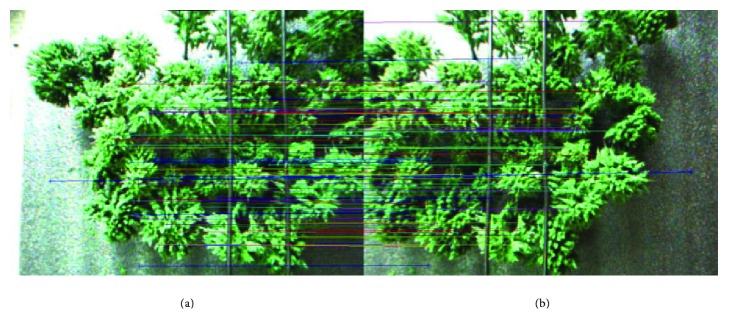
Image registration. (a) Original color image (left). (b) Original color image (right).

Traditional binocular image registration methods are slow and inaccurate. In the power line inspection system, because the point on the obstacle is connected, it is unnecessary to register all the pixels in the image. Based on the traditional SURF algorithm in [24], we make some optimizations in the matching strategy. Experiments show that the algorithm can effectively improve the speed and accuracy of image registration, which can be validated by the result in Figure 6. We can see that the selected feature points are randomly distributed in the image. The detailed procedure for image registration is as follows.

(1) Using SURF algorithm detects the feature points of the image in the scale-space. During the image filter processing, SURF algorithm selects a window with different sizes, and then the feature points are extracted by using Hessian matrix. And the point *D*(*x*, *y*) in the image is defined by Hessian matrix:

(3) $$\begin{array}{c} H\left(\;x,\sigma \;\right)=\left[\begin{array}{cc} L_{xx}\left(x,\sigma \right) & L_{xy}\left(x,\sigma \right)\\ L_{xy}\left(x,\sigma \right) & L_{yy}\left(x,\sigma \right) \end{array}\right], \end{array}$$

where *σ* varies in different scales and *L*_*xx*_(*x*, *σ*), *L*_*xy*_(*x*, *σ*), and *L*_*yy*_(*x*, *σ*) are defined as

(4) Lxxx,σ=Dx,y∗∂2∂x2gσ,Lxyx,σ=Dx,y∗∂2∂x∂ygσ,Lyyx,σ=Dx,y∗∂2∂y2gσ.

(2) Taking the current feature point as the center, a block of size 20*σ* is constructed along the main direction of the point. Then divide the area into 16 small areas, and calculate the Harr wavelet response in each 5*σ∗*5*σ* region. So each subarea can be represented by *v* = (∑*dx*, ∑|*dx*|, ∑*dy*, ∑|*dy*|). Finally, we obtain 64 dimensional descriptors of the point. The salient points in the image are defined as

(5) Pos1=x1′,y1′,x2′,y2′,…,xm′,ym′,1≤i≤m,Pos2=x1,y1,x2,y2,…,xn,yn,1≤i≤n,

where Pos1 represents the set of the feature points in the left image and Pos2 represents the set of the feature points in the right image.

(3) Calculate the Euclidean distance between all points in Pos1 and Pos2, and select the point with minimum Euclidean distance as the rough matching point. Sort the matching points in ascending order according to the Euclidean distance, and delete the abnormal points. Select the top *K* points in the matching points, which are defined as

(6) PosK=x1′,y1′,x1,y1,x2′,y2′,x2,y2,…,xK′,yK′,xK,yK.

(4) Next is screen matching points according to the slope between the corresponding points in Pos_*K*. In detail, firstly, calculate the frequency of each slope in all slopes, and the new slope set *k*_new = {*k*_1_, *k*_2_,…, *k*_*q*_} is formed by removing abnormal slopes. Secondly, a new set of matching points is defined as

(7) Pos_Knew=xz1,yz1,xy1,yy1,xz2,yz2,xy2,yy2,…,xzn,yzn,xyn,yyn.

### 4.4. Obstacle Detection

**Figure 7:**
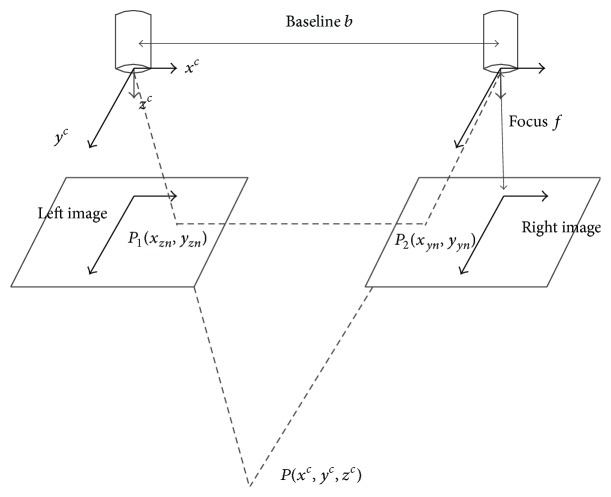
The principle of binocular vision imaging.

The purpose of using binocular vision camera is to precisely calculate distance information between external obstacles and the power lines. In this process, we get the 3D information of the obstacles and the power lines in 2D images according to characteristics of binocular vision as in [25]. The principle of binocular vision imaging is shown in Figure 7.

We already know the basic parameters of the binocular camera, such as the baseline *b* and the focus *f*. According to pos_*K*_new_, the parallax between left image and right image is defined as

(8) $$\begin{array}{c} d=\left(\;x_{zn}-x_{yn}\;\right). \end{array}$$

The spatial coordinates of a point* P* in the left camera coordinate system can be defined as

(9) xc=b·xznd,yc=b·yznd,zc=b·fd.

By calculating the spatial coordinates of all the feature points and the spatial coordinates of the points on the power line simultaneously, we judge whether the current point is a barrier by the minimum Euclidean distance *J* between the point and power lines. Finally, we deal with all the points and mark the points with less than the threshold value as obstacles and then enlarge the area of obstacles in a certain scale.

## 5. Evaluations and Discussions

**Figure 8:**
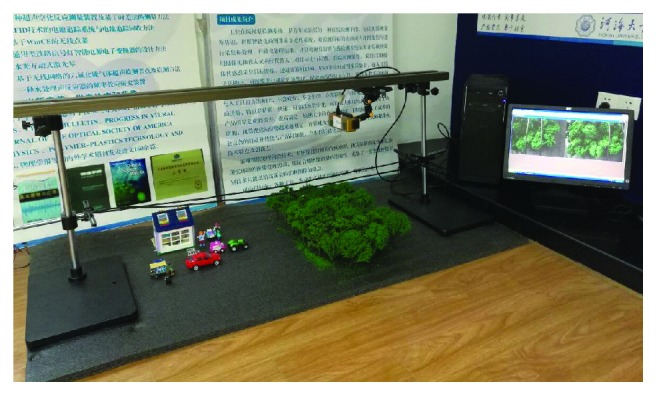
Experimental model of the power line inspection (the height of the obstacle models ranges from 20 to 150 mm and the height of the power line ranges from 150 to 155 mm.).

The object of the system in our work is to detect the external obstacles of power lines based on the human visual system model. In the practical application, we need to consider both the accuracy and the stability of the system under various conditions. In order to test the accuracy of the visual system, we set up the power line model in the laboratory. In this power line model, we have set up two power lines and complex background environment including trees and buildings. We can exactly measure the accuracy of the visual system according to the data we collected from the power line model. In addition, the various flight attitudes of the quadrotor helicopter are simulated in experiments with experimental model in Figure 8. In all experiments given in this paper, the time consumptions were measured on a standard PC Windows 8.1 running at Intel Core i7 2.5 GHz and 8 G RAM; the testing software was provided by MATLAB R2010a; the images are of size 384*∗*512.

### 5.1. Experiment of Normal Shooting under Forest-Based Environment

The experiment was carried out in a mixed environment consisting mainly of trees. During the normal flight of the quadrotor helicopter, power lines are nearly vertically distributed in images captured by binocular vision camera. In the process of analysis, firstly, we mark the external obstacles through manual measurement and record the measurement results in the corresponding table. And then we analyze the performance of the system through the comparison of the system data and the measured data.

In order to get the distance between an area and a power line, we usually first select a set of feature points within the area and then calculate the average distance of all these selected points with the power line. The accurate and false detection rates are defined as

**Figure 9:**
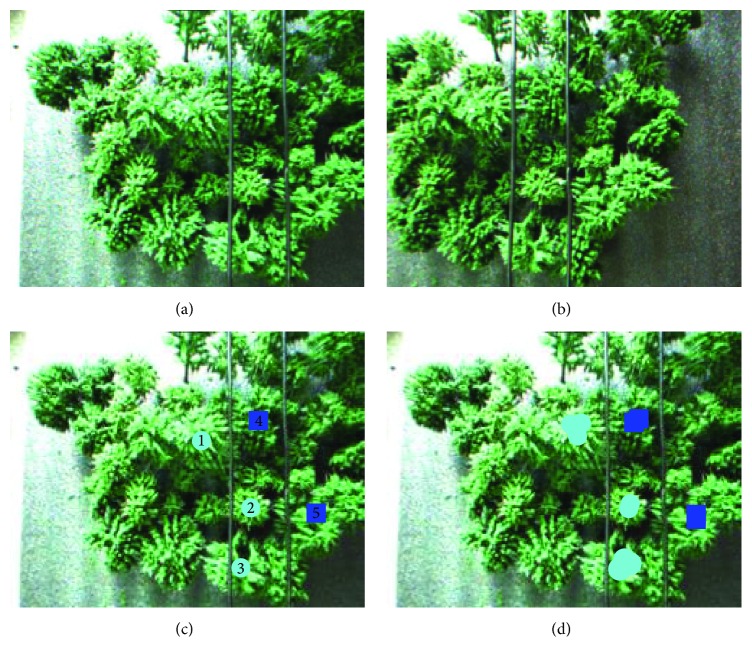
Obstacle detection (the marked circles are threats to the power line on the left, and the marked squares are threats to the power line on the left.). (a) Original image (left). (b) Original image (right). (c) Standard detection results (left). (d) Actual detection results (left).

(10) A=∑SE⊂SA∩SE≠0SA×100%,E=∑SE⊄SA∩SE≠0SE×100%,

where *S*_*A*_ are the actual obstacle connected domains and *S*_*E*_ are the experimental obstacle connected domains. In the experiment, a number of connected domains are used to describe a real obstacle area. However, if the connected domain is not corresponding to the obstacle, it will be judged as a false detection. From Example 1 (Figure 9 and Table 1), we can see that, in the case of normal shooting, the system can accurately complete the obstacle detection work, and the system accuracy rate is 100%.

**Figure 10:**
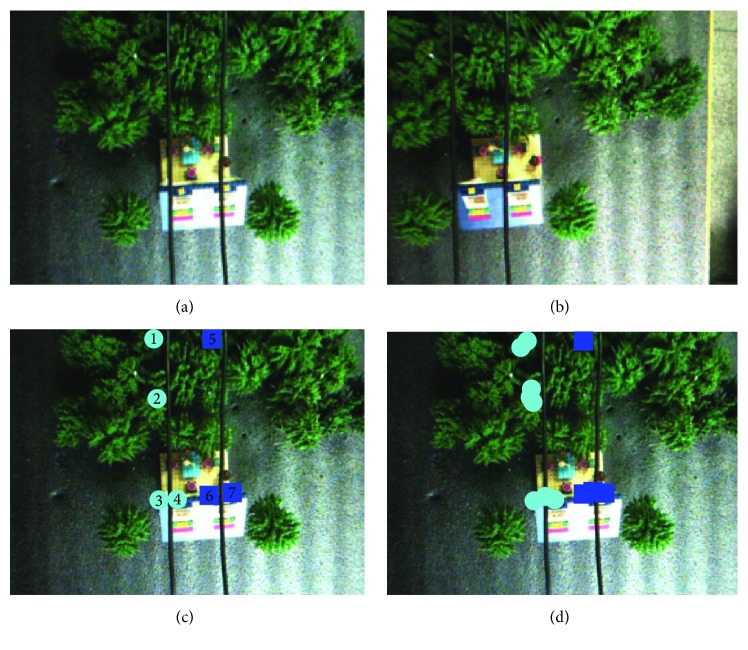
Obstacle detection. (a) Original image (left). (b) Original image (right). (c) Standard detection results (left). (d) Actual detection results (left).

In Example 2 (Figure 10 and Table 2), we have transformed the background of the experiment, and more power line obstacles appeared in the experiment. In this case, the system is still able to accurately output obstacle areas, and the accuracy rate is 100%.

The above experiments can validate the fact that our proposed power line obstacle detection method is feasible. In a variety of environmental experiments, our method has performed very well under normal shooting. The advantages of the system are mainly embodied in the less error, high accuracy, and low false detection rate.

**Example 1 .**

See Figure 9 and Table 1.

**Example 2 .**

See Figure 10 and Table 2.

### 5.2. Experiment of Abnormal Shooting under Building-Based Environment

The experiment was carried out in a mixed environment consisting mainly of flyovers. And the abnormal situations mainly act in three aspects. Firstly, the direction of shooting angle may deviate from the power lines distribution. Secondly, the attitude of the quadrotor helicopter may be tilted in the horizontal direction. Thirdly, both above-depicted circumstances show up simultaneously. In the working process of the quadrotor helicopter, such three factors as the staff's working state, the weather conditions, and the abnormal situations must be taken into consideration. However, these anomalies cannot be avoided completely in the actual work, motivating us to formulate such a system to deal with a variety of conditions effectively. Experiments show that the proposed method is effective for the images under abnormal shooting.

**Figure 11:**
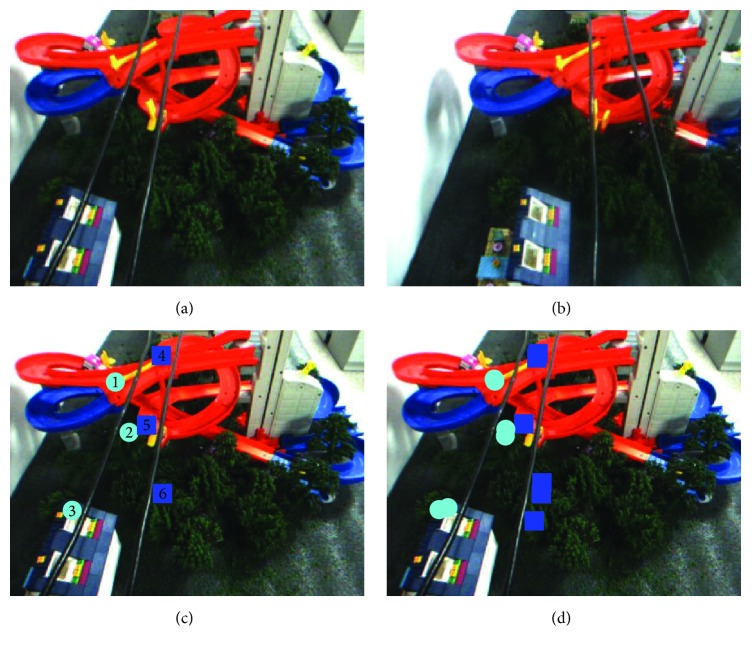
Obstacle detection. (a) Original image (left). (b) Original image (right). (c) Standard detection results (left). (d) Actual detection results (left).

From Example 3 (Figure 11 and Table 3), it can be seen that the direction of shooting angle is deviated from the power lines distribution. The experimental results show that the measurement error of the system is very small, although the phenomenon of false detection has been detected. Overall, our proposed method is stable in this condition.

**Figure 12:**
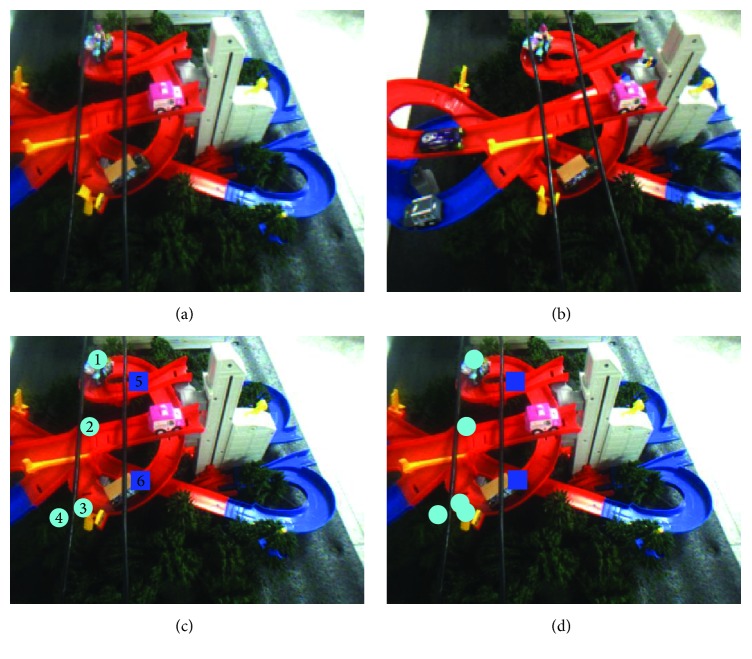
Obstacle detection. (a) Original image (left). (b) Original image (right). (c) Standard detection results (left). (d) Actual detection results (left).

From Example 4 (Figure 12 and Table 4), it can be found that the attitude of the quadrotor helicopter is tilted in the horizontal direction based on the distance difference between the power lines. The experimental results show that the system is stable in this condition without any loss of accuracy rate.

**Figure 13:**
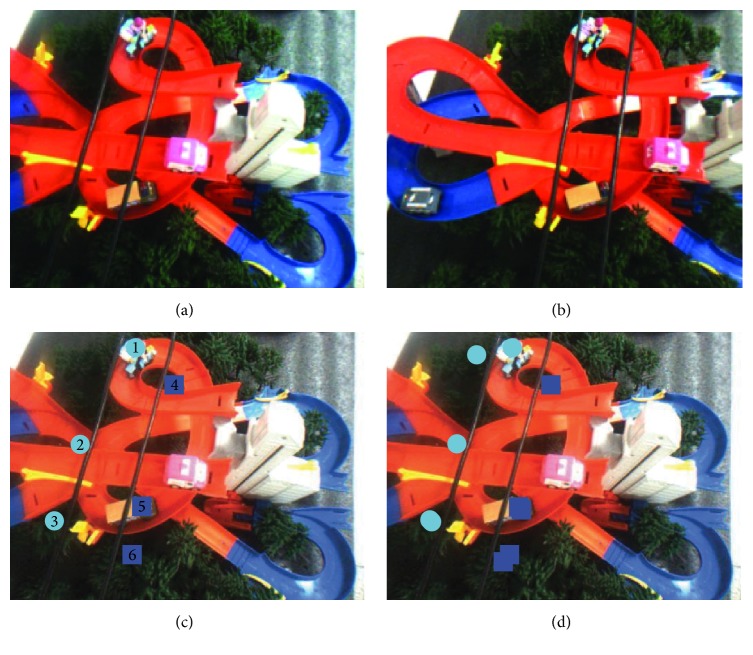
Obstacle detection. (a) Original image (left). (b) Original image (right). (c) Standard detection results (left). (d) Actual detection results (left).

In Example 5 (Figure 13 and Table 5), it can be seen that the direction of shooting angle is deviated from the power line distribution and the attitude of the quadrotor helicopter is tilted in the horizontal direction simultaneously. In this condition, the phenomenon of false detection has occurred in our system. The false detection rate of Example 5 is similar to that of Example 3. Therefore it can be concluded that the direction of shooting angle is deviating from the power line distribution and constitutes the main reason for false detection in the system. In order to verify this conclusion, we have done many experiments.

**Example 3 .**

See Figure 11 and Table 3.

**Example 4 .**

See Figure 12 and Table 4.

**Example 5 .**

See Figure 13 and Table 5.

Through many experiments, the results of Table 6 can prove our conclusion. Therefore, in the process of image acquisition, we need to try to keep the direction of shooting angle parallel to the direction of power line distribution. Although the system has the phenomenon of false detection, we can keep the detection accuracy of the obstacles in the connected area to be over 85%. Under normal circumstances, our proposed method can accurately detect the position of the power line external obstacles with the error range about 1 mm, and there is no missing detection. From the experimental results, we can see that each frame image processing time is about 2.5 s, which can basically meet the requirements of single frame image processing. However, the performance is not good enough in the processing of image sequences, which is to be solved in our future research work.

Many methods have been proposed for power line detection. In [26], a novel technique based on magnetic field sensing is proposed for underground power cable detection and inspection. This innovative algorithm provided good results, but it is not suitable for the air power line detection. In [4], the most important achievements in the field of power line inspection by mobile robots were presented. Although flying robots and climbing robots indeed have specific benefits, both of them are far from practical use. More attention to the control and communication of the UAV was paid in [27], but no solution was given to the power line detection. An unmanned aerial system based on the quadrotor helicopter for high voltage power line inspection was proposed in [5], but it just offered a simple solution of the power line using infrared and visible images. In this paper, we present a system for quantitative analysis of power line obstacles. The experimental results show that the proposed system can be applied to the actual power line inspection with high accuracy.

## 6. Conclusion

In this paper, we propose a power line inspection system based on the human visual system model. Compared with the traditional power line inspection systems, we introduce the characteristics of human vision into our system. In both the forest-based environment and the building-based environment, our system can not only detect the external obstacles of the power lines but also accurately measure the distance between the obstacles and the power lines. Moreover, our system provides a safer and cheaper power line inspection method. The system is of great importance to the electric power department and can greatly increase the efficiency and accuracy of power line inspection.

In the future, the proposed system is to be tested in a real complex environment. Besides, the adaptability of our system in abnormal situations is expecting further improvements. Still, our future work can focus on making the system more comprehensive, integrating optical cameras, infrared cameras, and ultraviolet cameras, and detecting faults within the power lines.

## Figures and Tables

**Table 1:** Experimental analysis.

	Area 1	Area 2	Area 3	Area 4	Area 5
Actual distance	11.8 mm	26.5 mm	5.1 mm	32.4 mm	10.5 mm
Experimental distance	13 mm	25.6 mm	5.3 mm	29.8 mm	9.5 mm
Error	1.2 mm	0.9 mm	0.2 mm	2.6 mm	1 mm

Accuracy rate: 100%; false detection rate: 0%; image processing time: 2.52 s					

**Table 2:** Experimental analysis.

	Area 1	Area 2	Area 3	Area 4	Area 5	Area 6	Area 7
Actual distance	12.4 mm	13.9 mm	5.1 mm	6.4 mm	8.2 mm	4.1 mm	6.5 mm
Experimental distance	13.5 mm	13.6 mm	6.7 mm	5.3 mm	7.7 mm	5.8 mm	6.3 mm
Error	1.1 mm	0.3 mm	1.6 mm	1.1 mm	0.5 mm	1.7 mm	0.2 mm

Accuracy rate: 100%; false detection rate: 0%; image processing time: 2.34 s							

**Table 3:** Experimental analysis.

	Area 1	Area 2	Area 3	Area 4	Area 5	Area 6
Actual distance	5.8 mm	13.4 mm	16.2 mm	5.3 mm	10.8 mm	15.6 mm
Experimental distance	6.3 mm	12.5 mm	15.7 mm	6.7 mm	9.2 mm	17.1 mm
Error	0.5 mm	0.9 mm	0.5 mm	1.4 mm	1.6 mm	1.5 mm

Accuracy rate: 100%; false detection rate: 14.3%; image processing time: 2.57 s						

**Table 4:** Experimental analysis.

	Area 1	Area 2	Area 3	Area 4	Area 5	Area 6
Actual distance	5.5 mm	6.9 mm	10.8 mm	15.5 mm	7.7 mm	14.6 mm
Experimental distance	6.7 mm	6.4 mm	11.2 mm	14.6 mm	6.8 mm	15.4 mm
Error	1.2 mm	0.5 mm	0.4 mm	0.9 mm	0.9 mm	0.8 mm

Accuracy rate: 100%; false detection rate: 0; image processing time: 2.44 s						

**Table 5:** Experimental analysis.

	Area 1	Area 2	Area 3	Area 4	Area 5	Area 6
Actual distance	5.5 mm	6.9 mm	15.5 mm	7.7 mm	14.6 mm	20.1 mm
Experimental distance	5.9 mm	5.6 mm	15.4 mm	6.8 mm	14.9 mm	19.4 mm
Error	0.4 mm	1.3 mm	0.1 mm	0.9 mm	0.3 mm	0.7 mm

Accuracy rate: 100%; false detection rate: 14.3%; image processing time: 2.47 s						

**Table 6:** Analysis of false detection.

	Test times under normal conditions/the number of false detection instances	Test times under abnormal conditions/the number of false detection instances
The direction of shooting angle	20/0	20/18
The attitude of the quadrotor helicopter	20/2	20/1
